# Anthropometric, cognitive, and schooling benefits of measles vaccination: Longitudinal cohort analysis in Ethiopia, India, and Vietnam

**DOI:** 10.1016/j.vaccine.2019.06.025

**Published:** 2019-07-18

**Authors:** Arindam Nandi, Anita Shet, Jere R. Behrman, Maureen M. Black, David E. Bloom, Ramanan Laxminarayan

**Affiliations:** a Center for Disease Dynamics, Economics & Policy, 1400 Eye St. NW, Suite 500, Washington, DC 20005, USA; b Johns Hopkins Bloomberg School of Public Health, 415 N. Washington Street, Baltimore, MD 21231, USA; c Economics Department, Perelman Center for Political Science and Economics, University of Pennsylvania, 133 South 36th Street, Philadelphia, PA 19104-6297, USA; d RTI International, Research Triangle Park, NC 27709, USA; e Department of Pediatrics, University of Maryland School of Medicine, 737 W. Lombard Street, Suite 161, Baltimore, MD 21201, USA; f Department of Global Health and Population, Harvard TH Chan School of Public Health, 665 Huntington Avenue, Building I 12th Floor, Suite 1202, Boston, MA 02115, USA; g Center for Disease Dynamics, Economics & Policy, B-25, Lajpat Nagar II, New Delhi, Delhi 110024, India; h Princeton Environmental Institute, Princeton University, Princeton, NJ 08544, USA

**Keywords:** Measles, India, Ethiopia, Vietnam, Young Lives, Long term effects

## Abstract

**Objective:**

To estimate the associations between measles vaccination and child anthropometry, cognition, and schooling outcomes in Ethiopia, India, and Vietnam.

**Methods:**

Longitudinal survey data from Young Lives were used to compare outcomes at ages 7–8 and 11–12 years between children who reported receipt or non-receipt of measles vaccine at 6–18 months-of-life (*n* = ∼2000/country). Z-scores of height-for-age (HAZ), BMI-for-age (BMIZ), weight-for-age (WAZ), Peabody Picture Vocabulary Test (PPVT), early grade reading assessment (EGRA), language and mathematics tests, and attained schooling grade were examined. Propensity score matching was used to control for systematic differences between measles-vaccinated and measles-unvaccinated children.

**Findings:**

Using age- and country-matched measles-unvaccinated children as comparisons, measles-vaccinated children had better anthropometrics, cognition, and schooling. Measles-vaccinated children had 0.1 higher HAZ in India and 0.2 higher BMIZ and WAZ in Vietnam at age 7–8 years, and 0.2 higher BMIZ at age 11–12 years in Vietnam. At ages 7–8 years, they scored 4.5 and 2.9 percentage points (pp) more on PPVT and mathematics, and 2.3 points more on EGRA in Ethiopia, 2.5 points more on EGRA in India, and 2.6 pp, 4 pp, and 2.7 points more respectively on PPVT, mathematics, and EGRA in Vietnam. At ages 11–12 years, they scored 3 pp more on English and PPVT in India, and 1.7 pp more on PPVT in Vietnam. They also attained 0.2–0.3 additional schooling grades across all ages and countries.

**Conclusion:**

Our findings suggest that measles vaccination may have benefits on cognitive gains and school-grade attainment that can have broad educational and economic consequences which extend beyond early childhood.

## 1 Introduction

An estimated 245,000 measles cases and 68,000 associated deaths occurred globally in 2016 [1]. Low- and middle-income countries (LMICs) bear the largest burden of the disease. In 2016, 50% of global measles cases and 30% of deaths occurred in India alone [1]. Measles has also resurged alarmingly in high-income countries in recent years [2], [3], [4], [5], [6], [7]. Outbreaks with hundreds of measles cases have been reported in Italy, Israel, Greece, France, Germany, and the USA [8], [9], [10].

The measles vaccine is a highly efficacious and cost-effective vaccine that prevented an estimated 21.1 million child deaths globally during 2000–2017 [11]. Recent observational, epidemiological modeling, and laboratory-based studies suggest additional benefits of the vaccine for children’s health [12]. Large-scale observational studies indicate that receipt of measles vaccine is associated with unexpectedly large reductions in measles-specific and all-cause childhood mortality [13].

In addition, the measles vaccine can affect long-term health and cognitive outcomes through two other pathways: first, via an indirect pathogen effect that prevents measles-associated functional immune suppression and immune amnesia [14] and, second, via the direct immunomodulatory effect of the live attenuated measles vaccine that induces innate immune memory and decreases incidence of nonmeasles infections [15], [16]. Because measles is the leading cause of blindness among children in low-income countries and can also cause hearing loss through ear infections and other complications such as pneumonia and diarrhea, immunization against the disease could improve educational outcomes in resource-poor settings [17], [18].

Specific evidence on links between measles vaccinations and schooling outcomes is based primarily on studies from Bangladesh and South Africa. The phased introduction of measles vaccine among 35,000 children in Bangladesh is associated with 7.4% higher school enrolment among boys but not girls [19]. A study of 4783 siblings in South Africa found that children receiving the measles vaccine attained an average of 0.2 more schooling grades compared with their measles-unvaccinated siblings [20]. However, both studies suffer from some important shortcomings. The Bangladesh study used a binary indicator of exposure to community-level measles vaccination programs instead of actual receipt of vaccines and did not control for households’ socioeconomic status. Neither study evaluated child anthropometric outcomes or cognitive test scores. Furthermore, these studies’ results may not be generalizable to other LMICs where underlying socioeconomic conditions, burdens of vaccine-preventable diseases, cognitive development, and schooling levels vary.

This study seeks to bridge this knowledge gap of specific associations between early-life receipt of measles vaccines and child anthropometry, cognition, and schooling attainment in three LMICs: Ethiopia, India, and Vietnam. The associations were examined among 7–12-year-old children using the Young Lives survey data from these countries [21]. All three countries are measles-endemic, with a total of 139,000 cases and 28,000 deaths in 2016, of which 87% and 72% respectively occurred in India [1].

## 2 Methods

### 2.1 Data: longitudinal Young Lives surveys

Young Lives, a publicly available study of the causes and consequences of childhood poverty, has followed ∼12,000 children in four LMICs—Ethiopia, India, Peru, and Vietnam—over 15 years [21]. In 2002, each country sample enrolled a “younger cohort” of ∼2000 children aged 6–18 months and an “older cohort” of ∼1000 children aged 7.5–8.5 years. Both cohorts were longitudinally resurveyed in 2006, 2009, 2013, and 2016. The younger cohort was used in this study.

Details have been published on the methodology, sampling strategy, participant characteristics, and data collected in Young Lives [22]. Children in poor households were overrepresented in 20 sentinel sites nationwide in Ethiopia, Peru, and Vietnam, and in the states of Andhra Pradesh and Telangana in India. Within each sentinel site, 100 households with children in the younger cohort were selected randomly. More than 98% of the selected households agreed to participate. Attrition rates between the baseline and the 2013 follow-up were very low, ranging from 2.2% to 2.9%, and attributable mainly to outmigration, household moves, and refusals [23]. Community-level data on infrastructure, amenities, and environment, such as the availability of public and private healthcare providers, were collected separately.

In 2002, caregiver-reported data on child vaccination were collected and cross-validated with vaccination cards when available. The rates of cross-validation were not recorded. Questions covered whether the child had received the Bacillus Calmette–Guérin (BCG) and measles vaccines that are typically administered at birth and at 9–12 months, respectively. In India and Peru, information on the oral polio vaccine—usually given at birth—was also collected. Data on children’s ages at the time of vaccinations or receipts of other vaccines were not collected.

Vaccination data from the 2002 survey were combined with socioeconomic and child anthropometric, cognition, and schooling data from the 2009 and 2013 surveys in Ethiopia, India, and Vietnam. Due to differences in age schedule for measles vaccines between Peru and the other Young Lives countries, 65% of Peruvian children were reported in 2002 as measles-unvaccinated (i.e., yet to be vaccinated). Therefore, Peru was excluded from analysis.

**Fig. 1:**
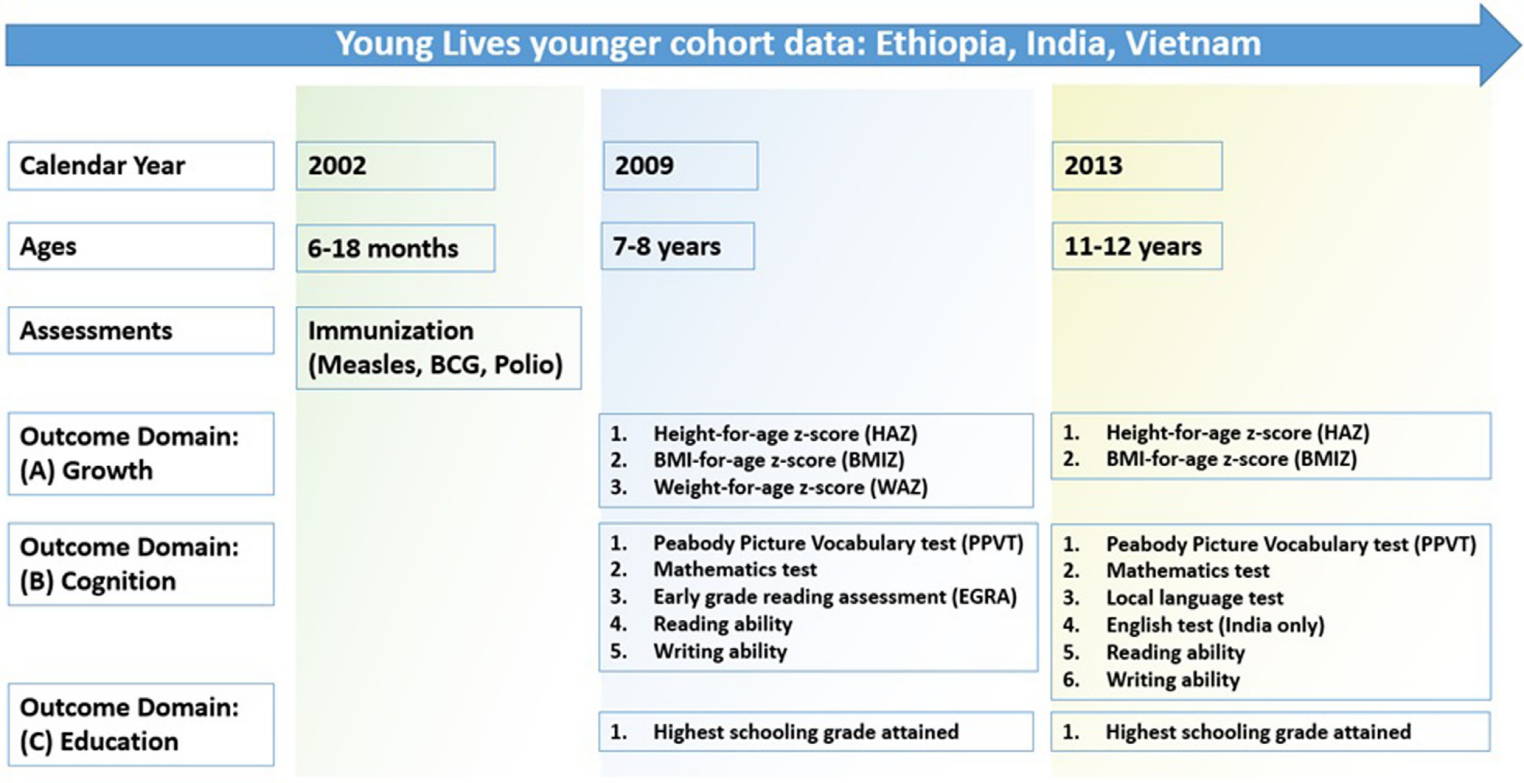
Selected Young Lives survey rounds and outcome indicators.

Using the 2009 survey data (children’s ages 7–8 years), the following anthropometric indicators were examined (Fig. 1): height-for-age z-scores (HAZ), body-mass-index-for-age z-scores (BMIZ), and weight-for-age z-scores (WAZ). Cognition indicators were based on percentage scores from all available standardized assessments in the surveys: Peabody Picture Vocabulary Test (PPVT), mathematics test, early-grade reading assessment (EGRA), and two binary measures (ability to read and write) [24], [25]. The schooling indicator was the highest grade attained at age 7–8 years.

These indicators were also examined using the 2013 data (children’s ages 11–12 years), with the following changes: WAZ, reading and writing ability, and EGRA were excluded because these data were not collected. Percentage local language test scores were included. In India only, percentage scores on an English language test were also available [24], [25].

The data collection process was uniform across the three countries. The surveyors made anthropometric measurements, conducted standardized cognition tests, and recorded scores. Children’s self-reported school grades attained were noted. Data on height and weight were converted into standardized z-scores [26]. Children with outlier values (HAZ < −6 or HAZ > 6, BMIZ < −5 or BMIZ > 5, and WAZ < −6 or WAZ > 5) were excluded (≤1% of sample).

The cognitive tests’ psychometric reliability and validity were discussed in a previous study [24]; that discussion is not repeated here. The PPVT consisted of age-appropriate questions adapted to each country. The mathematics test consisted of age-appropriate questions on counting, number recognition, and basic number operations. The 2009 EGRA test was drawn from the World Bank Living Standard Measurement Surveys [27] and involved reading and listening to words and passages in local languages and answering related questions [24]. The corrected global EGRA score, with a mean of 300 within each country, was analyzed. The 2013 local language and English tests involved reading words and passages and answering questions based on them. For the binary reading/writing ability test, the children were asked to read from literacy cards containing a few letters from the alphabet and one word and one sentence in local languages and to write another simple sentence consisting of three words. The binary variables indicated whether the child could fully read and write a sentence without difficulty, respectively. Highest schooling grades reported by children in the survey ranged from 0 to 4, 0 to 5, and 0 to 6 in 2009 and 1 to 8, 0 to 11, and 0 to 12 in 2013 in Ethiopia, India, and Vietnam, respectively.

### 2.2 Empirical methodology

Child vaccination rates in Ethiopia, India, and Vietnam vary substantially by region or subpopulation [28], [29], [30], [31], [32], [33], [34]. Households with access to vaccinations may be systematically different from those without access. Even when vaccines are accessible at low or no cost to households, cultural beliefs and perceptions, lack of awareness, transport costs, or child characteristics such as sex and perceived health status may affect parental decisions to vaccinate their children. For example, boys are vaccinated at much higher rates than girls in India even though there are no differences in access [35], [36].

Systematic biases that could arise because of differences between measles-vaccinated and measles-unvaccinated children were mitigated by using quasi-experimental propensity score matching (PSM) [37], [38], [39], [40]. First, within each country and survey round (2009 or 2013), the binary indicator of measles-vaccinated status was regressed on its potential determinants. These included child and household characteristics drawn from the concurrent survey round: child’s age in months, age in months squared, whether female, whether child was born prematurely, rural, household size, indicators of social or ethnic groups and religion and standard of living, age and sex of household head, and age and indicators of schooling attainment of the child’s mother and father. Supplementary webappendix section A contains the complete list of covariates and methodological details of PSM.

Based on the predicted probabilities of receiving measles vaccines (propensity scores) from this regression, measles-vaccinated children were matched with observationally similar measles-unvaccinated children within each country. If the covariates adequately determine measles-vaccination status, the differences in the outcome variable (known as average treatment effect on the treated, or ATT) between the two groups could be attributed to the measles vaccine. A kernel (Epanechnikov) matching algorithm was employed, and only observations with overlapping propensity scores (i.e., “common support”) were considered. To minimize the effect of attrition and missing values, matching was done separately for each outcome variable, survey round, and country sample.

The PSM model validity was examined through a series of tests of matching quality and alternative matching algorithms, along with sensitivity analyses on the subsample of children for whom data on healthcare access indicators were available. Supplementary webappendix section B presents the methodology and results. Supplementary webappendix section C discusses additional models comparing associations of measles vaccine vis-à-vis BCG vaccine (all countries) and polio vaccine (India).

## 3 Results

### 3.1 Study population characteristics

Table 1, Table 2, Table 3 present summary statistics of the Ethiopian, Indian, and Vietnamese samples, respectively. The first and third columns of Supplementary Table 1, Table 2, Table 3 also show the percentage differences in the raw (unmatched) data, i.e., differences in the mean values of child and household characteristics between measles-vaccinated and measles-unvaccinated groups, along with statistical significance.

**Table 1:** Summary statistics of Ethiopian children.

	2009 survey (7–8 year olds)		2013 survey (11–12 year olds)	
	Measles vaccinated	Measles unvaccinated	Measles vaccinated	Measles unvaccinated
	Mean ± SD	Mean ± SD	Mean ± SD	Mean ± SD
Height-for-age z-score (HAZ)	−1.2 ± 1.1	−1.3 ± 1.2	−1.5 ± 1	−1.5 ± 1
BMI-for-age z-score (BMIZ)	−1.3 ± 0.9	−1.2 ± 1	−1.8 ± 1	−1.8 ± 1
Weight-for-age z-score (WAZ)	−1.6 ± 1	−1.6 ± 0.9	–	–
PPVT percentage score (0–100)	42.9 ± 22.6	33.5 ± 19.2	71.4 ± 15.8	68.3 ± 15.8
Mathematics percentage score (0–100)	25.7 ± 19.6	18.7 ± 16.4	39.3 ± 22	35.1 ± 20.5
Language percentage score (0–100)	–	–	29.6 ± 15.1	25.9 ± 15.2
Global EGRA score	302.1 ± 16.1	296.8 ± 12.5	–	–
Highest schooling grade attained	0.8 ± 0.8	0.5 ± 0.7	3.8 ± 1.7	3 ± 1.7
Whether child can read	0.3 ± 0.5	0.2 ± 0.4	–	–
Whether child can write	0.2 ± 0.4	0.1 ± 0.3	–	–
Age of child in months	98.5 ± 3.5	96.1 ± 3.6	146.5 ± 3.7	144.1 ± 3.8
Whether child was born premature	0.1 ± 0.3	0.1 ± 0.3	0.1 ± 0.3	0.1 ± 0.3
Whether child is female	0.4 ± 0.5	0.5 ± 0.5	0.4 ± 0.5	0.5 ± 0.5
Rural household	0.5 ± 0.5	0.6 ± 0.5	0.5 ± 0.5	0.6 ± 0.5
Household size	6.2 ± 2	6.2 ± 2	5.8 ± 1.9	6 ± 1.9
Whether Amhara	0.2 ± 0.4	0.3 ± 0.5	0.2 ± 0.4	0.3 ± 0.5
Whether Oromo	0.2 ± 0.4	0.2 ± 0.4	0.2 ± 0.4	0.2 ± 0.4
Whether Tigrian	0.3 ± 0.5	0.1 ± 0.3	0.3 ± 0.5	0.1 ± 0.3
Whether Orthodox	0.7 ± 0.4	0.7 ± 0.5	0.7 ± 0.4	0.7 ± 0.5
Whether Muslim	0.2 ± 0.4	0.2 ± 0.4	0.2 ± 0.4	0.2 ± 0.4
Whether household head is female	0.2 ± 0.4	0.2 ± 0.4	0.3 ± 0.4	0.3 ± 0.4
Age of household head in years	44.2 ± 11.1	44.2 ± 11	46.9 ± 10.9	46.6 ± 10.2
Head’s schooling: literate but <6 years	0.2 ± 0.4	0.2 ± 0.4	0.2 ± 0.4	0.2 ± 0.4
Head’s schooling: ≥6 years but ≤11 years	0.2 ± 0.4	0.2 ± 0.4	0.2 ± 0.4	0.2 ± 0.4
Head’s schooling: ≥12 years	0.3 ± 0.5	0.3 ± 0.5	0.4 ± 0.5	0.6 ± 0.5
Mother’s schooling: literate but <6 years	0.2 ± 0.4	0.2 ± 0.4	0.1 ± 0.3	0.2 ± 0.4
Mother’s schooling: ≥6 years but ≤11 years	0.1 ± 0.4	0.1 ± 0.3	0.1 ± 0.4	0.1 ± 0.3
Mother’s schooling: ≥12 years	0.3 ± 0.4	0.2 ± 0.4	0.4 ± 0.5	0.4 ± 0.5
Mother’s age in years	34.3 ± 6.4	34.6 ± 6.3	38.3 ± 6.4	38.5 ± 6.3
Household belongs to wealth quintile 2	0.2 ± 0.4	0.2 ± 0.4	0.2 ± 0.4	0.2 ± 0.4
Household belongs to wealth quintile 3	0.2 ± 0.4	0.2 ± 0.4	0.2 ± 0.4	0.2 ± 0.4
Household belongs to wealth quintile 4	0.2 ± 0.4	0.2 ± 0.4	0.2 ± 0.4	0.2 ± 0.4
Household belongs to wealth quintile 5	0.2 ± 0.4	0.1 ± 0.3	0.2 ± 0.4	0.2 ± 0.4
Sample size	1,145	822	1,145	822

*Note:* Data are from 2009 to 2013 rounds of the Young Lives survey— about 2000 children in the younger cohort in Ethiopia. The sample is divided based on measles vaccination information from the 2002 round. SD denotes standard deviation. Empty cells indicate outcome variables not measured in that survey round.

**Table 2:** Summary statistics of Indian children.

	2009 survey (7–8 year olds)		2013 survey (11–12 year olds)	
	Measles vaccinated	Measles unvaccinated	Measles vaccinated	Measles unvaccinated
	Mean	Mean	Mean	Mean
Height-for-age z-score (HAZ)	−1.4 ± 1.1	−1.4 ± 1	−1.3 ± 1.4	−1.4 ± 1.3
BMI-for-age z-score (BMIZ)	−1.4 ± 1	−1.5 ± 1.1	−1.4 ± 1	−1.5 ± 1.1
Weight-for-age z-score (WAZ)	−1.8 ± 1.1	−1.9 ± 1	–	–
PPVT percentage score (0–100)	29.6 ± 15.3	26.2 ± 13.6	76.6 ± 13.3	72.5 ± 14.6
Mathematics percentage score (0–100)	42.7 ± 22.3	38.1 ± 21.5	44.9 ± 22.7	41.5 ± 22.7
Language percentage score (0–100)	–	–	56.7 ± 18.7	53.3 ± 18.3
Global EGRA score (0–100)	301.3 ± 15.4	296.5 ± 13.2	–	–
English percentage score	–	–	63.0 ± 19.9	58.7 ± 19.9
Highest schooling grade attained	1.8 ± 1	1.4 ± 0.9	5.5 ± 1.3	5.1 ± 1.3
Whether child can read	0.5 ± 0.5	0.4 ± 0.5	–	–
Whether child can write	0.4 ± 0.5	0.3 ± 0.5	–	–
Age of child in months	96.6 ± 3.6	94 ± 3.7	144.5 ± 3.6	142 ± 3.8
Whether child was born premature	0.1 ± 0.3	0.1 ± 0.3	0.1 ± 0.3	0.1 ± 0.3
Whether child is female	0.5 ± 0.5	0.5 ± 0.5	0.5 ± 0.5	0.5 ± 0.5
Rural household	0.7 ± 0.5	0.7 ± 0.5	0.7 ± 0.5	0.7 ± 0.5
Household size	5.4 ± 2.3	5.4 ± 2	4.8 ± 1.8	5 ± 1.8
Whether scheduled caste (SC)	0.2 ± 0.4	0.2 ± 0.4	0.2 ± 0.4	0.2 ± 0.4
Whether scheduled tribe (ST)	0.1 ± 0.3	0.2 ± 0.4	0.1 ± 0.3	0.2 ± 0.4
Whether other backward classes (OBC)	0.4 ± 0.5	0.5 ± 0.5	0.4 ± 0.5	0.5 ± 0.5
Whether Muslim	0.1 ± 0.3	0.1 ± 0.2	0.1 ± 0.3	0.1 ± 0.2
Whether Christian	0.1 ± 0.2	0 ± 0.2	0.1 ± 0.2	0 ± 0.2
Whether Buddhist	0 ± 0.1	0 ± 0.1	0 ± 0.1	0 ± 0.1
Whether household head is female	0.1 ± 0.3	0.1 ± 0.3	0.1 ± 0.3	0.1 ± 0.3
Age of household head in years	38.8 ± 9.3	38 ± 8.9	41.1 ± 7.6	41.6 ± 8.8
Head’s schooling: literate but <6 years	0.1 ± 0.3	0.1 ± 0.2	0.2 ± 0.4	0.2 ± 0.4
Head’s schooling: ≥6 years but ≤11 years	0.2 ± 0.4	0.2 ± 0.4	0.3 ± 0.5	0.2 ± 0.4
Head’s schooling: ≥12 years	0.3 ± 0.5	0.3 ± 0.5	0.3 ± 0.5	0.3 ± 0.5
Mother’s schooling: literate but <6 years	0.1 ± 0.3	0.1 ± 0.3	0.2 ± 0.4	0.1 ± 0.3
Mother’s schooling: ≥6 years but ≤11 years	0.3 ± 0.4	0.2 ± 0.4	0.3 ± 0.4	0.2 ± 0.4
Mother’s schooling: ≥12 years	0.1 ± 0.3	0.1 ± 0.3	0.2 ± 0.4	0.2 ± 0.4
Mother’s age in years	30.7 ± 4.3	30.3 ± 4.2	34.7 ± 4.3	34.3 ± 4.2
Household belongs to wealth quintile 2	0.2 ± 0.4	0.2 ± 0.4	0.2 ± 0.4	0.2 ± 0.4
Household belongs to wealth quintile 3	0.2 ± 0.4	0.2 ± 0.4	0.2 ± 0.4	0.2 ± 0.4
Household belongs to wealth quintile 4	0.2 ± 0.4	0.2 ± 0.4	0.2 ± 0.4	0.2 ± 0.4
Household belongs to wealth quintile 5	0.2 ± 0.4	0.2 ± 0.4	0.2 ± 0.4	0.2 ± 0.4
Sample size	1,462	549	1,462	549

*Note:* Data are from 2009 to 2013 rounds of the Young Lives survey—about 2000 children in the younger cohort in India. The sample is divided based on measles vaccination information from the 2002 round. SD denotes standard deviation. Empty cells indicate outcome variables not measured in that survey round.

**Table 3:** Summary statistics of Vietnamese children.

	2009 survey (7–8 year olds)		2013 survey (11–12 year olds)	
	Measles vaccinated	Measles unvaccinated	Measles vaccinated	Measles unvaccinated
	Mean	Mean	Mean	Mean
Height-for-age z-score (HAZ)	−0.6 ± 1.3	−0.7 ± 1.3	−0.6 ± 1.3	−0.7 ± 1.3
BMI-for-age z-score (BMIZ)	−1 ± 1	−1.2 ± 1.1	−1 ± 1.1	−1.1 ± 1.2
Weight-for-age z-score (WAZ)	−1.1 ± 1.3	−1.2 ± 1.3	–	–
PPVT percentage score (0–100)	48.4 ± 13.7	42.6 ± 13.3	77.8 ± 9.8	75.4 ± 12
Mathematics percentage score (0–100)	71 ± 19.2	59.7 ± 19.9	49 ± 16.5	46.7 ± 17
Language percentage score (0–100)	–	–	50.6 ± 16.5	48.2 ± 17.2
Global EGRA score	302.3 ± 13.8	296.6 ± 15.5	–	–
Highest schooling grade attained	1.9 ± 0.5	1.4 ± 0.6	5.8 ± 0.7	5.2 ± 1.1
Whether child can read	0.9 ± 0.3	0.8 ± 0.4	–	–
Whether child can write	0.9 ± 0.3	0.8 ± 0.4	–	–
Age of child in months	98.2 ± 3.4	94.8 ± 3.6	147.5 ± 3.2	144 ± 3.6
Whether child was born premature	0.1 ± 0.3	0.1 ± 0.3	0.1 ± 0.3	0.1 ± 0.3
Whether child is female	0.5 ± 0.5	0.5 ± 0.5	0.5 ± 0.5	0.5 ± 0.5
Rural household	0.7 ± 0.4	0.8 ± 0.4	0.7 ± 0.5	0.8 ± 0.4
Household size	4.6 ± 1.3	4.7 ± 1.5	4.5 ± 1.3	4.6 ± 1.5
Whether Kinh	0.9 ± 0.3	0.8 ± 0.4	0.9 ± 0.3	0.8 ± 0.4
Whether without any religion	0.9 ± 0.3	0.8 ± 0.4	0.9 ± 0.3	0.8 ± 0.4
Whether household head is female	0.1 ± 0.3	0.1 ± 0.3	0.1 ± 0.4	0.1 ± 0.3
Age of household head in years	42.1 ± 11.9	40.7 ± 12.1	44.3 ± 10.7	43.1 ± 10.3
Head’s schooling: literate but <6 years	0.1 ± 0.3	0.1 ± 0.3	0.2 ± 0.4	0.2 ± 0.4
Head’s schooling: ≥6 years but ≤11 years	0.2 ± 0.4	0.2 ± 0.4	0.5 ± 0.5	0.5 ± 0.5
Head’s schooling: ≥12 years	0.6 ± 0.5	0.5 ± 0.5	0.3 ± 0.4	0.2 ± 0.4
Mother’s schooling: literate but <6 years	0.1 ± 0.3	0.1 ± 0.3	0.2 ± 0.4	0.2 ± 0.4
Mother’s schooling: ≥6 years but ≤11 years	0.3 ± 0.4	0.2 ± 0.4	0.5 ± 0.5	0.5 ± 0.5
Mother’s schooling: ≥12 years	0.5 ± 0.5	0.4 ± 0.5	0.2 ± 0.4	0.2 ± 0.4
Mother’s age in years	34.4 ± 5.7	33.7 ± 5.8	38.4 ± 5.7	37.8 ± 5.8
Household belongs to wealth quintile 2	0.2 ± 0.4	0.2 ± 0.4	0.2 ± 0.4	0.2 ± 0.4
Household belongs to wealth quintile 3	0.2 ± 0.4	0.2 ± 0.4	0.2 ± 0.4	0.2 ± 0.4
Household belongs to wealth quintile 4	0.2 ± 0.4	0.2 ± 0.4	0.2 ± 0.4	0.2 ± 0.4
Household belongs to wealth quintile 5	0.2 ± 0.4	0.2 ± 0.4	0.2 ± 0.4	0.2 ± 0.4
Sample size	1,240	581	1,240	581

*Note:* Data are from 2009 to 2013 rounds of the Young Lives survey— about 2000 children in the younger cohort in Vietnam. The sample is divided based on measles vaccination information from the 2002 round. SD denotes standard deviation. Empty cells indicate outcome variables not measured in that survey round.

Among the ∼2000 children in each country, 58.2%, 72.7%, and 68.1% in Ethiopia, India, and Vietnam, respectively, were reported as measles-vaccinated in 2002. In comparison, the national measles vaccination rates among 12–23-month-old children were 36% and 56% respectively in Ethiopia and India in 2002 [41]. The official measles vaccination rate among 12–23-month-old Vietnamese children was 96% in 2002, but household survey–based estimates indicate the rate to have been between 75% and 83% [42]. Among the poorest quintile of Vietnamese children—i.e., those overrepresented in the Young Lives surveys—the rate was estimated to be 52%–70% in 2002 [42].

As seen in the 2009 and 2013 surveys, measles-vaccinated children were 2–3 months older than measles-unvaccinated children in all countries. In Ethiopia and Vietnam, children from households in the highest wealth quintile received measles vaccination at significantly higher rates than children in other wealth quintiles. Statistically significant differences in measles vaccination were also evident across social, ethnic, and religious groups.

### 3.2 Associations of measles vaccinations and child anthropometrics

Table 4 presents the estimated associations of measles vaccination obtained from PSM analysis (ATT). The estimates are changes in z-scores. In 2009, measles-vaccinated children had 0.13 (95% CI: 0, 0.26; p < 0.05) higher HAZ in India and 0.18 (95% CI: 0, 0.36; p < 0.05) higher BMIZ and 0.23 (95% CI: 0.04, 0.4; p < 0.05) higher WAZ in Vietnam, as compared with matched measles-unvaccinated children from the same countries. In 2013, measles-vaccinated Vietnamese children had 0.19 (95% CI: 0, 0.37; p < 0.05) higher BMIZ than matched measles-unvaccinated children.

**Table 4:** Estimated anthropometric, cognitive, and schooling associations (ATT) of measles vaccine among children in Ethiopia, India, and Vietnam.

	Ethiopia		India		Vietnam	
	ATT	p-value	ATT	p-value	ATT	p-value
*2009 survey (7–8 year olds):*						
Height-for-age z-score (HAZ)	0.10	0.16	0.13	0.05	0.14	0.06
BMI-for-age z-score (BMIZ)	−0.09	0.17	0.08	0.23	0.18	0.04
Weight-for-age z-score (WAZ)	0.02	0.76	0.12	0.07	0.23	0.01
PPVT percentage score (0–100)	4.47	0.00	1.28	0.15	2.59	0.01
Mathematics percentage score (0–100)	2.87	0.01	−0.07	0.96	4.02	0.00
Global EGRA score	2.29	0.03	2.47	0.01	2.71	0.01
Whether child can read	0.08	0.00	0.04	0.21	0.04	0.11
Whether child can write	0.07	0.00	0.06	0.05	0.06	0.03
Highest schooling grade attained	0.15	0.00	0.23	0.00	0.21	0.00

*2013 survey (11–12 year olds):*						
Height-for-age z-score (HAZ)	0.06	0.34	0.10	0.13	0.11	0.22
BMI-for-age z-score (BMIZ)	0.03	0.69	0.10	0.24	0.19	0.04
English percentage score (0–100)	–	–	3.11	0.01	–	–
PPVT percentage score (0–100)	1.32	0.22	3.30	0.00	1.66	0.05
Language percentage score (0–100)	1.89	0.08	2.01	0.08	0.95	0.46
Mathematics percentage score (0–100)	2.44	0.10	2.46	0.08	0.42	0.74
Highest schooling grade attained	0.29	0.01	0.23	0.00	0.21	0.01

*Note:* Data are from 2009 to 2013 rounds of the Young Lives survey— about 2000 children in the younger cohort in each country. ATT denotes the propensity score matching estimator (kernel matching) of the association of measles vaccination. Empty cells indicate outcome variables not measured in that survey round. Depending upon the country and survey round, some values of the outcome indicators were missing.

### 3.3 Associations of measles vaccinations and cognitive test scores

In 2009, measles-vaccinated children in Ethiopia received 4.47 percentage points (pp) (95% CI: 1.96, 6.96; p < 0.001), 2.87 pp (95% CI: 0.67, 5.07; p < 0.05), and 2.29 points (95% CI: 0.26, 4.3; p < 0.05) higher scores respectively on PPVT, mathematics test, and EGRA than matched measles-unvaccinated children. They were also 8 pp (95% CI: 3, 13; p < 0.01) and 7 pp (95% CI: 2, 11; p < 0.01) more likely to be able to read and write respectively in their native languages than the matched control group. In India, measles-vaccinated children scored 2.47 points (95% CI: 0.72, 4.22; p < 0.01) more on EGRA and were 6 pp (95% CI: 0, 12; p < 0.05) more likely to be able to write in their native languages as compared with matched measles-unvaccinated children. In Vietnam, measles-vaccinated children scored 2.59 pp (95% CI: 0.66, 4.52; p < 0.01), 4.02 pp (95% CI: 1.22, 6.81; p < 0.01), and 2.71 points (95% CI: 0.53, 4.89; p < 0.05) more on PPVT, mathematics test, and EGRA, respectively, than matched measles-unvaccinated children. They also were 6 pp (95% CI: 0, 10; p < 0.05) more likely to be able to write in their native languages.

In 2013, measles-vaccinated Indian children scored 3.11 pp (95% CI: 0.65, 5.55; p < 0.05) and 3.3 pp (95% CI: 1.56, 5.04; p < 0.001) more on the English test and PPVT, respectively, as compared with matched measles-unvaccinated children. In Vietnam, measles-vaccinated children scored 1.66 pp (95% CI: 0, 3.32; p < 0.05) higher on PPVT than the matched control group.

### 3.4 Associations of measles vaccinations and schooling

In 2009, measles-vaccinated children attained 0.2 (95% CI: 0.05, 0.23; p < 0.01) more schooling grades in Ethiopia, 0.2 (95% CI: 0.11, 0.34; p < 0.001) more grades in India, and 0.2 (95% CI: 0.12, 0.29; p < 0.001) more grades in Vietnam, as compared with matched measles-unvaccinated children.

In 2013, measles-vaccinated children attained 0.3 (95% CI: 0.07, 0.5; p < 0.01) more schooling grades in Ethiopia, 0.2 (95% CI: 0.07, 0.38; p < 0.01) more grades in India, and 0.2 (95% CI: 0.06, 0.35; p < 0.01) more grades in Vietnam, as compared with matched measles-unvaccinated children.

## 4 Discussion and conclusion

Using longitudinal data from Ethiopia, India, and Vietnam, we found that children who received measles vaccinations when they were 6–18 months of age had better anthropometrics, performed better in standardized cognitive tests, and completed more schooling grades in some cases when they were 7–12 years of age than similar children who had not received measles vaccinations. Measles-vaccinated children had better weight scores (WAZ) in India and Vietnam and better height scores (HAZ) in India, compared with measles-unvaccinated children. Measles-vaccinated Ethiopian children scored higher on PPTV, mathematics, and reading and writing tests than measles-unvaccinated children. Measles-vaccinated Indian children scored higher in EGRA, writing, PPVT, and English tests than measles-unvaccinated children. Measles-vaccinated Vietnamese children scored higher in the PPVT, EGRA, mathematics, and writing tests as compared with measles-unvaccinated children.

The schooling grade attainment associations with measles vaccines were consistently positive and statistically significant at ages 7–8 and 11–12 years in all three countries. As discussed in supplementary webappendix section B, the findings were not sensitive to variations in propensity score matching methods. The results were similar, although weaker for ages 11–12 years, after including healthcare access indicators in the model. They were also similar – with more statistically significat positive associations – when we used a nearest-neighbor covariate matching method with exact macth on child age in the 2002 data.

Analysis of a vaccinated-only sample (supplementary webappendix section C) showed that the positive anthropometric, cognitive, and schooling attainment associations of measles vaccinations persisted in the additional models, although the effects were less prominent in the case of Ethiopia. This was expected because children who received neither measles nor BCG vaccine were excluded from this analysis. These unvaccinated children were likely to have worse anthropometric, cognitive, and schooling outcomes than children who received at least one vaccine.

These findings reinforce the limited literature on the longer-term benefits of measles vaccine based on studies from Bangladesh and South Africa [19], [20]. They are also relevant for a small but growing literature on the long-term, nonhealth benefits of vaccines in general, which includes studies of the maternal tetanus vaccine in Bangladesh, *Haemophilus influenzae* Type B (Hib) vaccination in India, and full child immunization in the Philippines [43], [44], [45]. Because early childhood development encompasses several factors including nutrition, infection, nurturing, and environmental exposure, the long-term associations of vaccination with child outcomes is difficult to establish. Well-characterized birth or childhood cohorts followed longitudinally for a decade or longer, such as Young Lives, are highly valuable in this context because time-invariant characteristics of households and individuals can be incorporated. Quasi-experimental methodologies such as PSM used in conjunction with such data can reduce additional biases arising from time-varying factors and provide robust estimates of the associations of vaccination.

The findings have important policy implications because they indicate that the benefits of vaccination are broader than those related to early-life mortality, morbidity, and healthcare costs. In 2010, stunting and poverty prevented 250 million under-5 children worldwide from realizing their development potential [46]. Recommendations for these children include “nurturing care”, which incorporates attention to their health, nutrition, learning opportunities, responsive caregiving, and safety and security [47], [48]. Through reduced measles burden, along with the potential anthropometric, cognitive, and schooling benefits, the measles vaccine may amplify the positive effects of the other compoenents of nurturing care over the life cycle.

The value of vaccines over the life course is increasingly recognized, and researchers have attempted to measure the impact of vaccines on health equity, financial risk protection, long-term health improvements, and reduction in antibiotic resistance [49], [50], [51]. The World Health Organization is currently developing an approach for systematically measuring the broader benefit of vaccines in the context of LMICs [52].

During 1980–2016, full immunization (diphtheria-tetanus-pertussis third dose, or DTP3) rates of children increased globally from 12% to 86% [53]. However, 19.5 million infants worldwide, primarily from heavily populated, low-income countries such as India, Indonesia, Nigeria, and Pakistan, remain partially vaccinated or unvaccinated [54]. In particular, 2.2 million and 0.44 million children remain unvaccinated in India and Ethiopia, respectively, while child vaccination rates are near-universal in Vietnam [54].

The recent resurgence of measles in high-income countries with historically low burden of the disease has also highlighted the need for the 95% or greater global vaccine coverage that is required for full protection of the population [7], [8], [9], [10], [55]. While international in-migration of susceptible individuals is a potential source of measles transmission [5], measles vaccination rates among children have dropped below the 95% coverage threshold for herd immunity in some high-income countries, e.g., 90% and 89% coverage in France and Italy, respectively, in 2016 [41].

The associations between measles vaccinations and cognitive and schooling outcomes could imply potential economic benefits. Each extra year of primary or secondary schooling is estimated to increase earnings by 6% in India and more in Ethiopia [56]. In Vietnam, one extra grade of schooling is estimated to increase earnings 2.7%–5%, depending upon the type of employment [57].

The analysis has some limitations. Although PSM mitigated the impacts of systemic differences in observed characteristics of measles-vaccinated and measles-unvaccinated children on the estimates, unobserved characteristics arguably could have affected the estimates. For example, parents may have decided to vaccinate based on the health status of children (i.e., prioritizing vaccination of children who were sick more often) or provide more schooling resources to certain children based on their innate capabilities, both of which were unobserved in the data. If such unobserved factors were linked with cognitive or schooling outcomes, PSM could not reduce the selection biases.

Children in the three study countries receive the first dose of measles vaccine at age 9–12 months after birth, although early and delayed vaccinations are common [58], [59]. Children under the age of 9 months could be given a supplementary dose of the measles vaccine, followed by the regular first dose at 9–12 months. The control group in our analysis contained both children who possibly never received measles vaccination in their lifetime and those who received it after the 2002 survey. Although the second Young Lives survey round (2006–2007) again collected information on children’s vaccination status, we did not use those data because of potential measurement error due to a lengthy recall period. The estimates associations in our study are for vaccination vis-à-vis nonvaccination and delayed vaccination for measles and therefore may be conservative. In additional analyses using a covariate matching method in which we matched children based on their exact age, the findings were similar.

Our study could not capture potential secondary immunity benefits of measles vaccines provided to other children within the same households or communities of the measles-vaccinated children due to lack of data. Therefore, our estimates of the associations between measles vaccinations and improved future outcomes are likely to be conservative.

In LMICs, the availability and quality of schooling may affect standardized cognitive test scores, schooling enrollment, and grade attained by children [60]. Systematic review studies show that the factors influencing children’s academic outcomes vary substantially across LMIC contexts [60]. In high-income countries where the variability in schooling inputs is lower, health interventions for polio, pneumonia, and infant care have been linked with increased schooling attainment [61], [62], [63], although there are no long-term studies of the measles vaccine.

Finally, a potential methodological limitation of the study concerns multiple hypotheses testing [64], [65], [66], which increases the probability of finding some significant associations by chance. However, we note that even under conservative adjustment methods [64], the main results generally would be significant.

Despite such limitations, our findings from three very different LMICs suggest that the benefits of measles vaccinations may extend well beyond early childhood and have broad educational and economic consequences that are often incompletely considered when investing in immunization programs.

## 5 Funding statement

This work was supported by the Bill & Melinda Gates Foundation [OPP1183738]. The funders had no role in study design, data collection and analysis, decision to publish, or preparation of the manuscript.

## Declaration of Competing Interest

DB has previously received personal fees from GlaxoSmithKline plc, Merck, Pfizer, and Sanofi-Pasteur related generally to value of vaccination research but not this study. All other authors declare no conflict of interest.
