# Supplementary material for: Anthropometric, cognitive, and schooling benefits of measles vaccination: Longitudinal cohort analysis in Ethiopia, India, and Vietnam
Source: Vaccine. 2019 Jul 18;37(31):4336–43. doi: 10.1016/j.vaccine.2019.06.025 (PMC6620502; doi:10.1016/j.vaccine.2019.06.025)
Supplement: Supplementary data 1 [file mmc1.docx]

**SUPPLEMENTARY WEBAPPENDIX**

**Section A: Methodological details of propensity score matching**

The following briefly describes the propensity score matching (PSM) methodology [1–4]:

Let $Y{}_{i1}$ and $Y_{i0}$ denote a later-life indicator (e.g., highest schooling grade attained) of child *i* for measles vaccination receipt (treatment) and nonreceipt (control). Let the indicator *M*=1 if the child received measles vaccination, and *M*=0 otherwise. Then, the effect of the treatment for a child is given by $\tau_{i}=(Y{}_{i1}-Y{}_{i0})$. The estimated average treatment effect on the treated (ATT) is defined as

$${\tau|}_{I=1}=E\left( \tau_{i} \right|M=1)=E\left( Y_{i1} \right|M=1)-E\left( Y_{i0} \right|M= 1)$$

For a given child, observing both the treatment and control outcomes $Y{}_{i1}$ and $Y_{i0}$ at the same time is not possible. Therefore, simple group comparisons are likely to produce biased estimates of the treatment’s effect.

Let us consider a covariate vector *X* that includes individual and household characteristics of study participants, e.g., age, sex, birth order, parental schooling attainment, social group, religion, and measures of household standard of living. Following the so-called *unconfoundedness* assumption[4], conditional upon the set *X,* the outcome variable is considered to be independent of the treatment status, i.e., $\left( Y{}_{i1} ,Y{}_{i0} \right)\perp(M\left| X \right).$

Now, let $\rho(X)=P(M=1|X)$ be the probability of being in the treatment group (received measles vaccine) conditional upon the covariates. $\rho(X)$ is known as the propensity score. Then, following the unconfoundedness assumption and the overlap assumption $[0<\rho(X)<1\forall X]$ of propensity scores [4], the later-life outcome of a child is orthogonal to treatment status conditional upon the estimated propensity score, i.e.,

$(Y{}_{i1} ,Y{}_{i0})\perp M|\rho(X)$.

Therefore,$E\left( Y_{1}-Y_{0} \right|M=1, p(X))=E(Y_{1}-Y_{0} | \rho(X))$. A matching estimator of ATT can be generated as

$$ATT= {\tau|}_{M=1}= E\left[ E\left( Yi \right|\rho\left( X_{i} \right), M_{i}=1 \right)-E\left( Y{}_{i} \right|\rho\left( X_{i} \right), M_{i}=0)|M_{i}=1]$$

In this equation, the first term on the right-hand side is the average outcome of children in the treatment group. The second term denotes the average outcome from a matched comparison group.

The PSM analysis was conducted separately using 2009 and 2013 survey data in each country, combined with vaccination information for each child from 2002. Within each country and survey round (2009 or 2013), the likelihood of receiving measles vaccination was regressed (probit model) on child and household characteristics from the concurrent survey round, including child’s age in months, age in months squared, sex (whether female), whether child was born prematurely, location (whether rural), household size, indicators of socioeconomic or ethnic groups and religion, age and sex of household head, and age and indicators of schooling attainment of the mother and father of the child. For Ethiopia, indicators of Amhara, Oromo, and Tigrian ethnic groups and indicators of Muslims and Orthodox Christians were included. For India, social group indicators for scheduled caste, scheduled tribe, and other backward classes and religion indicators for Muslims, Christians, and Buddhists were included. For Vietnam, indicators for the Kinh ethnic group and those with no religion were included.

Maternal and paternal schooling attainment was classified into four groups: no schooling, <6 years of schooling, ≥6 years but ≤11 years of schooling, and ≥12 years of schooling. The three highest-level indicators were included in the regression. Household standard of living was created with a composite index of ownership of durable assets such as television, radio, and car, along with household living conditions such as indicators of construction quality and access to toilets and electricity [5]. Indicators for the second to fourth quintiles of the index were included in the regression. The matching ATT estimator was estimated separately for each outcome indicator, survey round, and country combination. Additional models in which healthcare access indicators were also included are discussed in section B.

**Section B: Tests of matching quality, inclusion of healthcare access indicators, and use of alternative propensity score matching algorithm**

Matching quality was assessed by examining systematic differences in individuals between the measles-vaccinated and measles-unvaccinated groups. An indicator of quality was the standardized percentage bias, which was estimated separately in the unmatched and matched data. Bias is the difference of the sample means of a covariate between the two groups as a percentage of the square root of the average of the sample variances of the groups [6]. If matching quality is high, the mean bias should decline considerably post-matching. In another test, PSM was conducted and the subsample of only the matched observations from measles-vaccinated and measles-unvaccinated groups was taken. Then, the first-stage propensity score regression model was re-estimated on this subsample. If matching quality is high, the goodness of fit (R^2^) of this model should be substantially lower compared with the R^2^ of the original first-stage model [7].

Supplementary Tables 1, 2, and 3 present the tests of matching quality. Due to space limitations, we only present the HAZ analysis results. Models for other outcome indicators produced highly similar results.

PSM successfully reduced systemic differences between the treatment and control groups in all three countries. In Ethiopia, the mean percentage bias in covariates decreased from 14 and 14.5 before matching to 3.1 and 2.8 after matching in the analysis of 2009 and 2013 data, respectively. The pseudo-R^2^ also decreased substantially from 0.16 and 0.2 pre-matching in 2009 and 2013 to 0.005 and 0.006 post-matching, respectively.

Similarly, the mean percentage bias decreased 58–74% post-matching in the Indian and Vietnamese data analyses. The post-matching pseudo-R^2^ values in these models were also less than a fifth of the pseudo-R^2^ values before matching.

**Inclusion of healthcare access indicators**

From Young Lives 2002 survey data, we obtained binary indicators of the availability of a public hospital, private hospital, public health center, family planning center, community health worker, and a midwife at the community level (village or city ward). These data were available for about 80% of children in Ethiopia and Vietnam and 82% of children in India. We repeated the original kernel PSM by including these indicators (along with all other covariates discussed in section A) in the first-stage probit regression model of PSM.

The results, presented in Supplementary Table 4, are similar to those from the base case model. Measles-vaccinated children in Ethiopia scored 2.71 percentage points (pp) and 2.25 pp more on PPVT and mathematics test, were 6% more likely to be able to write, and attained 0.2 extra schooling grades in 2009, as compared with measles-unvaccinated children.

In India, measles-vaccinated children scored 2.5 points higher on EGRA, were 7% more likely to be able to read, and attained 0.2 more schooling grades than the control group in 2009. In 2013, they scored 3.2 pp higher in PPVT.

In Vietnam, measles-vaccinated children scored 3.18 pp higher in PPVT and attained 0.2 extra schooling grades in 2009 as compared with measles-unvaccinated children. They also scored 1.81 pp more on PPVT and attained 0.3 extra schooling grades in 2013.

**Alternative propensity score matching algorithms**

The sensitivity of our results was tested by using two alternative matching methods. In the first, we used a kernel (Epanechnikov) matching algorithm with a logit model of propensity score estimation as the first stage of PSM. In the second, we used the original probit model of propensity scores but matched each treatment group observation with the three nearest neighbors (with replacement) in the control group.

Supplementary Tables 5, 6, and 7 present results from robustness checks with additional matching algorithms. They are similar to the findings from the base model. Measles-vaccinated children had 0.1 higher HAZ in India and 0.2 higher HAZ and BMIZ in Vietnam than matched measles-unvaccinated children in 2009. In Ethiopia, they received 4–5 pp more on PPVT, 3 pp more on the mathematics test, and 2 points more on EGRA and were 7–10 pp more likely to be able to read and write in 2009. In India, measles-vaccinated children scored 2.53 points more on EGRA and were 6 pp more likely to be able to write than the control group in 2009. They also scored 3–4 pp higher on PPVT in 2013. In Vietnam, measles-vaccinated children scored 4 pp higher on the mathematics test and 3 points higher on EGRA and were 6 pp more likely to be able to write than measles-unvaccinated children in 2009.

In all three countries and survey rounds, measles-vaccinated children attained 0.2–0.3 more schooling grades as compared with matched measles-unvaccinated children.

**Covariate matching analysis with exact matching on child’s age**

The recommended age for the first dose of measles vaccine in Ethiopia, India, and Vietnam is 9–12 months of life. Between 20% and 24% of children in our sample across the three countries were under the age of 9 months in 2002. Most of these children were reported in the data—and considered in our model—as measles-unvaccinated (i.e., potentially awaiting vaccination).

The remaining children reported receiving the measles vaccine possibly due to one or more of the following reasons. The WHO recommends a supplementary measles vaccine dose at age 6 months for infants living in, or traveling to, areas with a measles outbreak, and for immunosuppressed infants living in measles endemic areas [8]. These children should also be given the regular measles vaccine doses starting at age 9 months.

Early measles vaccination may also occur due to systemic inefficiencies, i.e., providers not following the recommended vaccination schedule [9]. A study in India used nationally representative survey data from 2008 and found that among 12-23-month-old children who received the first dose of the measles vaccine, 15% did so before 9 months [9]. Finally, vaccination data in the 2002 Young Lives surveys might have suffered from reporting errors.

To mitigate the effect of any measurement errors related to the age at measles vaccination, we conducted additional analyses using a covariate matching method [10]. We matched intervention and control group children based on the joint distribution of the same covariates (background characteristics of children) which were used in the first stage probit of the original PSM model. Each intervention observation was matched with its one-to-one nearest neighbor in the control group. We employed exact matching on the age of children in the 2002 data, i.e. a match for an intervention group child was obtained only from the subsample of the control group children with the same age (in months) as the intervention child. Standard errors were heteroskedasticity robust. The difference in outcome between the intervention child and matched control child was the ATT estimator of the association of measles vaccination.

Supplementary Table 8 shows the results, which were similar to the base case model. In 2009, measles-vaccinated children in Ethiopia had 0.15 higher HAZ, and they scored 7.51 pp more on PPVT, 6.78 pp more on mathematics, and 4.79 points more on EGRA tests than the control group. They were 11 pp and 10 pp more likely to be able to read and write than the control group, and also attained 0.3 extra schooling grades. In India, measles-vaccinated children scored 2.52 points higher on EGRA and attained 0.3 more schooling grades that the control group. In Vietnam, they had 0.33 and 0.36 higher BMIZ and WAZ respectively, and were 9 pp more likely to be able to read.

In 2013, measles-vaccinated Ethiopian children scored 3.47 pp more on PPVT, were 3.18 pp and 4.03 pp more likely to be able to read and write, and attained 0.7 more schooling grades as compared with the control group. Measles-vaccinated Indian children had 0.16 higher HAZ, and scored 5.97 pp, 3.69 pp, 4.05 pp, and 4.76 pp more on English, PPVT, language, and mathematics tests respectively than the control group. In Vietnam, measles-vaccinated children had 0.19 higher HAZ and 0.27 higher BMIZ, and attained 0.1 more schooling grades as compared with the control group.

**Supplementary Table 1: Tests of matching quality in the analysis of Ethiopian data**

|  | 2009 survey  (7–8 year olds) | | 2013 survey  (11–12 year olds) | |
| --- | --- | --- | --- | --- |
|  | % bias before matching | % bias after matching | % bias before matching | % bias after matching |
| Age of child in months | 68.2 | 0.2 | 80.6 | 1.2 |
| Squared age of child in months | 67.9 | 0.2 | 80.5 | 1.2 |
| Whether child was born premature | 1.9 | -2.8 | -2.1 | -5.3 |
| Whether child is female | -9.3 | -1.1 | -11.9 | -0.2 |
| Rural household | -15.4 | -10.6 | -5.5 | -2.7 |
| Household size | -4.4 | -4.7 | -7.1 | -7.5 |
| Whether Amhara | -20.2 | 3.6 | -33.4 | -4.6 |
| Whether Oromo | -10.1 | -4.2 | -3 | -1.7 |
| Whether Tigrian | 54.3 | -0.8 | 59.9 | -2.4 |
| Whether Orthodox | 11.8 | -0.3 | 11.5 | -2 |
| Whether Muslim | -1.5 | -4 | -1.6 | -0.7 |
| Whether household head is female | 11.5 | 2.9 | 3 | 3.3 |
| Age of household head in years | 0.7 | -0.8 | 0.5 | 0.8 |
| Head’s schooling: < 6 years | -8 | 2.6 | 1.3 | -1.6 |
| Head’s schooling: ≥ 6 years but ≤ 11 years | 1.6 | 3.1 | 2.9 | -0.5 |
| Head’s schooling: ≥ 12 years | 1.3 | 2.2 | -6.2 | 2.3 |
| Mother’s schooling: < 6 years | -9 | 3.8 | -1.6 | 2.2 |
| Mother’s schooling: ≥ 6 years but ≤ 11 years | 12.2 | 2.4 | 2.5 | -1.8 |
| Mother’s schooling: ≥ 12 years | 2 | 1.2 | -6.4 | 5.5 |
| Mother’s age in years | -4 | -3.7 | 5.7 | 1.7 |
| Household belongs to wealth quintile 2 | -1.9 | -8.3 | 3.4 | 3.9 |
| Household belongs to wealth quintile 3 | -0.2 | 2.3 | -4.2 | -5 |
| Household belongs to wealth quintile 4 | 3 | 0.8 | 4.8 | 4.3 |
| Household belongs to wealth quintile 5 | 16.3 | 8.6 | 8.4 | -3.6 |
| Mean % bias | 14.0 | 3.1 | 14.5 | 2.8 |
| Pseudo R^2^ | 0.16 | 0.005 | 0.2 | 0.006 |

Note: Data are from 2009 and 2013 rounds of the Young Lives survey— about 2,000 children in the younger cohort in Ethiopia. Bias is the standardized percentage difference in the value of a variable between the measles-vaccinated and measles-unvaccinated children. Matching was based on propensity scores, using a kernel matching method.

**Supplementary Table 2: Tests of matching quality in the analysis of Indian data**

|  | 2009 survey  (7–8 year olds) | | 2013 survey  (11–12 year olds) | |
| --- | --- | --- | --- | --- |
|  | % bias before matching | % bias after matching | % bias before matching | % bias after matching |
| Age of child in months | 69.3 | -0.2 | 66.3 | -0.9 |
| Squared age of child in months | 69.1 | -0.3 | 66.1 | -1 |
| Whether child was born premature | 4.8 | 1.7 | 4.8 | 0.6 |
| Whether child is female | 2.5 | -2.1 | 2.5 | -0.2 |
| Rural household | -1.9 | -6.9 | -10.1 | -7.9 |
| Household size | -0.5 | -1.5 | -10.3 | -3.2 |
| Whether scheduled caste (SC) | 7.2 | -8.2 | 7.2 | -8.3 |
| Whether scheduled tribe (ST) | -7 | -0.8 | -7 | -1.6 |
| Whether other backward classes (OBC) | -9.9 | 1.3 | -9.9 | 2.5 |
| Whether Muslim | 5.8 | 5.5 | 5.8 | 3.4 |
| Whether Christian | 7.2 | 0 | 7.2 | 2.2 |
| Whether Buddhist | 2.6 | -0.8 | 2.6 | 3.8 |
| Whether household head is female | -6.7 | -0.1 | -6.7 | 0.5 |
| Age of household head in years | 9 | -3.1 | -6.2 | -1 |
| Head’s schooling: < 6 years | 12.1 | 4.7 | 0.8 | -1 |
| Head’s schooling: ≥ 6 years but ≤ 11 years | 10.6 | 3 | 9.5 | 0.3 |
| Head’s schooling: ≥ 12 years | -4.1 | 0.4 | -1.6 | 5.6 |
| Mother’s schooling: < 6 years | 4.6 | -0.6 | 4.8 | -3.1 |
| Mother’s schooling: ≥ 6 years but ≤ 11 years | 9.7 | 5.6 | 13.7 | 9.6 |
| Mother’s schooling: ≥ 12 years | 1.8 | 5.9 | -3.2 | 2 |
| Mother’s age in years | 9.3 | 2.4 | 9.3 | 3.5 |
| Household belongs to wealth quintile 2 | -6.5 | -9.4 | -3.3 | -5.9 |
| Household belongs to wealth quintile 3 | -0.5 | -1 | -5.1 | 2.7 |
| Household belongs to wealth quintile 4 | -6.3 | 0.1 | 2 | -1.1 |
| Household belongs to wealth quintile 5 | 18.1 | 9.1 | 15.3 | 5.5 |
| Mean % bias | 11.5 | 3.0 | 11.3 | 3.1 |
| Pseudo R^2^ | 0.10 | 0.006 | 0.09 | 0.006 |

Note: Data are from 2009 and 2013 rounds of the Young Lives survey—about 2,000 children in the younger cohort in India. Bias is the standardized percentage difference in the value of a variable between the measles-vaccinated and measles-unvaccinated children. Matching was based on propensity scores, using a kernel matching method.

**Supplementary Table 3: Tests of matching quality in the analysis of Vietnamese data**

|  | 2009 survey  (7–8 year olds) | | 2013 survey  (11–12 year olds) | |
| --- | --- | --- | --- | --- |
|  | % bias before matching | % bias after matching | % bias before matching | % bias after matching |
| Age of child in months | 103.6 | -4.3 | 102.6 | -8.7 |
| Squared age of child in months | 103.1 | -4.8 | 101.7 | -8.9 |
| Whether child was born premature | -5.5 | 9 | 2.3 | 8.6 |
| Whether child is female | -6.1 | -4.2 | -1.9 | 1.2 |
| Rural household | -21.8 | -14.6 | -20 | -12.8 |
| Household size | -0.1 | -3.6 | -12.1 | -5.3 |
| Whether Kinh | 4.2 | 5.1 | 19.9 | 5.8 |
| Whether without any religion | 10.8 | 4.3 | 5.8 | 4.4 |
| Whether household head is female | 2.2 | 7.1 | 9.2 | 5 |
| Age of household head in years | 10.9 | -1.7 | 11.4 | 5.3 |
| Head’s schooling: < 6 years | -7.8 | -4.4 | -4 | -5.7 |
| Head’s schooling: ≥ 6 years but ≤ 11 years | 1.9 | -2 | 1.3 | -0.8 |
| Head’s schooling: ≥ 12 years | 10.1 | 13.9 | 12.1 | 10.1 |
| Mother’s schooling: < 6 years | -4.9 | -6.7 | -1.7 | -9.8 |
| Mother’s schooling: ≥ 6 years but ≤ 11 years | 3.7 | -1 | 12.1 | -0.4 |
| Mother’s schooling: ≥ 12 years | 8.5 | 7.3 | 6.4 | 11 |
| Mother’s age in years | 14 | 8.2 | 10.9 | 6.6 |
| Household belongs to wealth quintile 2 | -3.8 | -13.8 | -1.2 | -0.9 |
| Household belongs to wealth quintile 3 | -3.7 | -6.4 | -0.7 | -1.9 |
| Household belongs to wealth quintile 4 | -0.4 | 4.7 | 4.1 | -2.4 |
| Household belongs to wealth quintile 5 | 14.9 | 16 | 15.4 | 11.2 |
| Mean % bias | 16.3 | 6.8 | 17.0 | 6.0 |
| Pseudo R^2^ | 0.19 | 0.019 | 0.22 | 0.013 |

Note: Data are from 2009 and 2013 rounds of the Young Lives survey—about 2,000 children in the younger cohort in Vietnam. Bias is the standardized percentage difference in the value of a variable between the measles-vaccinated and measles-unvaccinated children. Matching was based on propensity scores, using a kernel matching method.

**Supplementary Table 4: Estimated anthropometric, cognitive, and schooling** **associations (ATT) of measles vaccine among children in Ethiopia, India, and Vietnam, incorporating healthcare access indicators**

|  | Ethiopia | | India | | Vietnam | |
| --- | --- | --- | --- | --- | --- | --- |
|  | ATT | p-value | ATT | p-value | ATT | p-value |
| *2009 survey (7–8 year olds):* |  |  |  |  |  |  |
| Height-for-age z-score (HAZ) | 0.05 | 0.49 | 0.08 | 0.25 | 0.13 | 0.11 |
| BMI-for-age z-score (BMIZ) | -0.10 | 0.11 | 0.06 | 0.39 | 0.09 | 0.31 |
| Weight-for-age z-score (WAZ) | -0.02 | 0.77 | 0.09 | 0.21 | 0.16 | 0.09 |
| PPVT percentage score (0–100) | 3.25 | 0.01 | 0.99 | 0.29 | 3.36 | 0.00 |
| Mathematics percentage score (0–100) | 2.17 | 0.05 | -0.02 | 0.99 | 3.22 | 0.04 |
| Global EGRA score | 1.86 | 0.11 | 2.35 | 0.02 | 1.93 | 0.11 |
| Whether child can read | 0.06 | 0.04 | 0.01 | 0.69 | 0.04 | 0.11 |
| Whether child can write | 0.06 | 0.00 | 0.06 | 0.06 | 0.05 | 0.06 |
| Highest schooling grade attained | 0.17 | 0.00 | 0.22 | 0.00 | 0.19 | 0.00 |
|  |  |  |  |  |  |  |
| *2013 survey (11–12 year olds):* |  |  |  |  |  |  |
| Height-for-age z-score (HAZ) | 0.08 | 0.27 | 0.06 | 0.43 | 0.12 | 0.21 |
| BMI-for-age z-score (BMIZ) | 0.01 | 0.84 | 0.06 | 0.48 | 0.18 | 0.08 |
| English percentage score (0–100) | -- | -- | 2.60 | 0.06 | -- | -- |
| PPVT percentage score (0–100) | 1.46 | 0.26 | 3.26 | 0.00 | 2.00 | 0.03 |
| Language percentage score (0–100) | 1.15 | 0.35 | 0.91 | 0.47 | 0.89 | 0.54 |
| Mathematics percentage score (0–100) | 1.45 | 0.39 | 2.03 | 0.20 | 0.44 | 0.75 |
| Highest schooling grade attained | 0.28 | 0.02 | 0.16 | 0.07 | 0.25 | 0.00 |

Note: Data are from 2009 and 2013 rounds of the Young Lives survey—about 2,000 children in the younger cohort in in each country. ATT denotes the propensity score matching estimator (kernel matching) of the association of measles vaccination. Empty cells indicate outcome variables not measured in that survey round. Depending upon the country and survey round, some values of the outcome indicators were missing.

**Supplementary Table 5: Robustness check—estimated benefits of measles vaccination in Ethiopia using alternative matching methods**

|  | Kernel matching with logit model | | Three-nearest-neighbors matching | |
| --- | --- | --- | --- | --- |
|  | ATT | p-value | ATT | p-value |
| *2009 survey (7–8 year olds):* |  |  |  |  |
| Height-for-age z-score (HAZ) | 0.11 | 0.15 | 0.12 | 0.06 |
| BMI-for-age z-score (BMIZ) | -0.09 | 0.18 | -0.07 | 0.27 |
| Weight-for-age z-score (WAZ) | 0.02 | 0.73 | 0.01 | 0.81 |
| PPVT percentage score (0–100) | 4.45 | 0.00 | 5.04 | 0.00 |
| Mathematics percentage score (0–100) | 2.86 | 0.01 | 3.29 | 0.01 |
| Global EGRA score | 2.29 | 0.03 | 2.28 | 0.07 |
| Whether child can read | 0.08 | 0.00 | 0.10 | 0.00 |
| Whether child can write | 0.07 | 0.00 | 0.08 | 0.00 |
| Highest schooling grade attained (0–4) | 0.14 | 0.00 | 0.18 | 0.00 |
|  |  |  |  |  |
| *2013 survey (11–12 year olds):* |  |  |  |  |
| Height-for-age z-score (HAZ) | 0.06 | 0.32 | 0.08 | 0.16 |
| BMI-for-age z-score (BMIZ) | 0.03 | 0.68 | -0.01 | 0.90 |
| PPVT percentage score (0–100) | 1.32 | 0.22 | 1.74 | 0.11 |
| Language percentage score (0–100) | 1.84 | 0.09 | 1.44 | 0.22 |
| Mathematics percentage score (0–100) | 2.42 | 0.10 | 1.97 | 0.17 |
| Highest schooling grade attained (1–8) | 0.28 | 0.01 | 0.24 | 0.03 |

Note: Data are from 2009 and 2013 rounds of the Young Lives survey—about 2,000 children in the younger cohort in Ethiopia. ATT denotes the propensity score matching estimator of the association of measles vaccination. The sample sizes of measles-vaccinated and measles-unvaccinated groups are 1,145 and 822, respectively, with minor variation across PSM models due to missing values.

**Supplementary Table 6: Robustness check—estimated benefits of measles vaccination in India using alternative matching methods**

|  | Kernel matching with logit model | | Three-nearest-neighbors matching | |
| --- | --- | --- | --- | --- |
|  | ATT | p-value | ATT | p-value |
| *2009 survey (7–8 year olds):* |  |  |  |  |
| Height-for-age z-score (HAZ) | 0.13 | 0.04 | 0.13 | 0.05 |
| BMI-for-age z-score (BMIZ) | 0.08 | 0.25 | 0.09 | 0.24 |
| Weight-for-age z-score (WAZ) | 0.12 | 0.07 | 0.10 | 0.20 |
| PPVT percentage score (0–100) | 1.33 | 0.14 | 0.37 | 0.78 |
| Mathematics percentage score (0–100) | 0.06 | 0.97 | -1.14 | 0.54 |
| Global EGRA score | 2.53 | 0.00 | 2.14 | 0.08 |
| Whether child can read | 0.04 | 0.20 | 0.02 | 0.66 |
| Whether child can write | 0.06 | 0.04 | 0.03 | 0.44 |
| Highest schooling grade attained (0–5) | 0.24 | 0.00 | 0.19 | 0.00 |
|  |  |  |  |  |
| *2013 survey (11–12 year olds):* |  |  |  |  |
| Height-for-age z-score (HAZ) | 0.10 | 0.12 | 0.09 | 0.15 |
| BMI-for-age z-score (BMIZ) | 0.10 | 0.24 | 0.07 | 0.49 |
| English percentage score (0–100) | 3.27 | 0.01 | 2.64 | 0.09 |
| PPVT percentage score (0–100) | 3.33 | 0.00 | 3.86 | 0.01 |
| Language percentage score (0–100) | 2.08 | 0.07 | 2.17 | 0.11 |
| Mathematics percentage score (0–100) | 2.55 | 0.07 | 1.94 | 0.26 |
| Highest schooling grade attained (0–11) | 0.23 | 0.00 | 0.29 | 0.01 |

Note: Data are from 2009 and 2013 rounds of the Young Lives survey—about 2,000 children in the younger cohort in India. ATT denotes the propensity score matching estimator of the association of measles vaccination. The sample sizes of measles-vaccinated and measles-unvaccinated groups are 1,462 and 549, respectively, with minor variation across PSM models due to missing values.

**Supplementary Table 7: Robustness check—estimated benefits of measles vaccination in Vietnam using alternative matching methods**

|  | Kernel matching with logit model | | Three-nearest-neighbors matching | |
| --- | --- | --- | --- | --- |
|  | ATT | p-value | ATT | p-value |
| *2009 survey (7–8 year olds):* |  |  |  |  |
| Height-for-age z-score (HAZ) | 0.16 | 0.04 | 0.12 | 0.14 |
| BMI-for-age z-score (BMIZ) | 0.19 | 0.04 | 0.18 | 0.04 |
| Weight-for-age z-score (WAZ) | 0.24 | 0.01 | 0.23 | 0.01 |
| PPVT percentage score (0–100) | 2.70 | 0.01 | 1.48 | 0.13 |
| Mathematics percentage score (0–100) | 3.96 | 0.01 | 3.70 | 0.01 |
| Global EGRA score | 2.82 | 0.01 | 2.86 | 0.02 |
| Whether child can read | 0.04 | 0.09 | 0.04 | 0.13 |
| Whether child can write | 0.06 | 0.02 | 0.05 | 0.06 |
| Highest schooling grade attained (0–6) | 0.21 | 0.00 | 0.21 | 0.00 |
|  |  |  |  |  |
| *2013 survey (11–12 year olds):* |  |  |  |  |
| Height-for-age z-score (HAZ) | 0.12 | 0.15 | 0.11 | 0.25 |
| BMI-for-age z-score (BMIZ) | 0.20 | 0.03 | 0.13 | 0.21 |
| PPVT percentage score (0–100) | 1.79 | 0.03 | 1.22 | 0.15 |
| Language percentage score (0–100) | 1.10 | 0.39 | 0.65 | 0.65 |
| Mathematics percentage score (0–100) | 0.63 | 0.62 | 0.93 | 0.51 |
| Highest schooling grade attained (0–12) | 0.22 | 0.00 | 0.20 | 0.01 |

Note: Data are from 2009 and 2013 rounds of the Young Lives survey—about 2,000 children in the younger cohort in Vietnam. ATT denotes the propensity score matching estimator of the association of measles vaccination. The sample sizes of measles-vaccinated and measles-unvaccinated groups are 1,240 and 581, respectively, with minor variation across PSM models due to missing values.

**Supplementary Table 8: Estimated anthropometric, cognitive, and schooling** **associations (ATT) of measles vaccine in Ethiopia, India,** **and Vietnam, using covariate matching method**

|  | Ethiopia | | India | | Vietnam | |
| --- | --- | --- | --- | --- | --- | --- |
|  | ATT | p-value | ATT | p-value | ATT | p-value |
| *2009 survey (7–8 year olds):* |  |  |  |  |  |  |
| Height-for-age z-score (HAZ) | 0.15 | 0.03 | 0.07 | 0.45 | 0.20 | 0.08 |
| BMI-for-age z-score (BMIZ) | -0.04 | 0.60 | 0.07 | 0.41 | 0.33 | 0.00 |
| Weight-for-age z-score (WAZ) | 0.09 | 0.14 | 0.07 | 0.37 | 0.36 | 0.00 |
| PPVT percentage score (0–100) | 7.51 | 0.00 | 1.18 | 0.34 | 1.89 | 0.20 |
| Mathematics percentage score (0–100) | 6.78 | 0.00 | 0.11 | 0.95 | 2.75 | 0.15 |
| Global EGRA score | 4.79 | 0.00 | 2.52 | 0.03 | 1.97 | 0.19 |
| Whether child can read | 0.11 | 0.00 | 0.01 | 0.74 | 0.09 | 0.00 |
| Whether child can write | 0.10 | 0.00 | 0.06 | 0.11 | 0.03 | 0.48 |
| Highest schooling grade attained | 0.27 | 0.00 | 0.25 | 0.00 | 0.08 | 0.28 |
|  |  |  |  |  |  |  |
| *2013 survey (11–12 year olds):* |  |  |  |  |  |  |
| Height-for-age z-score (HAZ) | 0.02 | 0.74 | 0.16 | 0.03 | 0.19 | 0.04 |
| BMI-for-age z-score (BMIZ) | 0.13 | 0.07 | 0.13 | 0.16 | 0.27 | 0.03 |
| English percentage score (0–100) | -- | -- | 5.97 | 0.00 | -- | -- |
| PPVT percentage score (0–100) | 3.47 | 0.00 | 3.69 | 0.00 | 1.75 | 0.27 |
| Language percentage score (0–100) | 3.18 | 0.01 | 4.05 | 0.00 | -0.01 | 1.00 |
| Mathematics percentage score (0–100) | 4.03 | 0.01 | 4.76 | 0.01 | -0.22 | 0.89 |
| Highest schooling grade attained | 0.65 | 0.00 | 0.23 | 0.05 | 0.12 | 0.02 |

Note: Data are from 2009 and 2013 rounds of the Young Lives survey—about 2,000 children in the younger cohort in in each country. Those under the age of 9 months in 2002 were excluded. ATT denotes the nearest neighbor covariate matching estimator of the association of measles vaccination. Empty cells indicate outcome variables not measured in that survey round. Depending upon the country and survey round, some values of the outcome indicators were missing.

**Section C: Estimated benefits of measles vaccine as compared with BCG and polio vaccines**

**Methods**

The routine childhood vaccines in our study countries include polio, measles, BCG, and DPT, following the World Health Organization recommendation [11]. All of these vaccines were scheduled to be administered within the first 14 weeks of birth, except for measles, which was to be first given at 9 months. The 2002 Young Lives survey collected data on measles and BCG vaccines in Ethiopia and Vietnam and on measles, BCG, and polio vaccines in India.

For this exploration, we restricted the study sample to children who received BCG vaccine: 73.5%, 92.4%, and 88.9% of all children in Ethiopia, India, and Vietnam, respectively. Then, we divided these children into two groups: those who received BCG but not measles vaccine and those who received both vaccines. Among children who received BCG, 77.9%, 71.4%, and 69.4% in these countries also received the measles vaccine. We used the PSM method (kernel matching) to compare the health, cognitive, and schooling outcomes of the children who received both vaccines with those of similar children who received only BCG vaccine within each country and survey round. The difference in outcomes can be attributed to the measles vaccine.

For India, we repeated the analysis by restricting the sample to children who received the polio vaccine (95.4% of all children). Of these, 75.9% also received the measles vaccine. We used PSM (kernel matching) to compare the outcomes of children who received both measles and polio vaccines with similar children who only received polio vaccine.

**Results**

Supplementary Table 9 shows the estimated associations of measles vaccination as compared with other vaccines. Only estimates that are statistically significant at 5% are discussed.

In Vietnam, measles- and BCG-vaccinated children had 0.3 standard deviations higher BMIZ and WAZ scores in 2009 and 0.3 higher BMIZ scores in 2013, as compared with BCG-only-vaccinated children. Measles- and BCG-vaccinated Indian children had 0.2 higher BMIZ scores than BCG-only-vaccine children in 2013. No health benefits were seen in Ethiopia.

With respect to BCG, the measles vaccine was also associated with 4.25 pp higher PPVT in Ethiopia, 2.73 pp higher PPVT and 2.04 points higher EGRA in India, and 3.67 pp higher PPVT, 4.95 pp higher mathematics, and 2.52 points higher EGRA scores in Vietnam in 2009. In 2013, the measles vaccine—with respect to BCG—was associated with 4.06 pp higher PPVT in India and 1.79 pp higher PPVT and 2,.71 higher language scores in Vietnam.

With respect to the polio vaccine, the measles vaccine in India was associated with 2.2 pp higher PPVT and 2.42 points higher Global EGRA scores and 7 pp higher writing ability in 2009. It was also associated with 3.05 pp higher English and 3.62 pp higher PPVT scores in 2013.

In both BCG-only and polio-only models, the measles vaccine was associated with 0.1–0.3 additional grades of schooling in all country and survey rounds.

**Supplementary Table 9: Estimated associations of measles vaccination in comparison with other vaccines**

|  | Ethiopia: measles vaccine compared with BCG | | India: measles vaccine compared with BCG | | India: measles vaccine compared with polio | | Vietnam: measles vaccine compared with BCG | |
| --- | --- | --- | --- | --- | --- | --- | --- | --- |
|  | ATT | p-value | ATT | p-value | ATT | p-value | ATT | p-value |
| *2009 survey (7–8 year olds):* |  |  |  |  |  |  |  |  |
| Height-for-age z-score (HAZ) | -0.05 | 0.62 | 0.06 | 0.43 | 0.12 | 0.11 | 0.13 | 0.13 |
| BMI-for-age z-score (BMIZ) | -0.08 | 0.39 | 0.07 | 0.35 | 0.07 | 0.34 | 0.27 | 0.01 |
| Weight-for-age z-score (WAZ) | -0.10 | 0.26 | 0.07 | 0.38 | 0.10 | 0.16 | 0.28 | 0.01 |
| PPVT percentage score (0–100) | 4.25 | 0.03 | 2.73 | 0.01 | 2.20 | 0.02 | 3.67 | 0.00 |
| Mathematics percentage score (0–100) | 2.56 | 0.11 | 0.35 | 0.83 | 0.76 | 0.62 | 4.95 | 0.00 |
| Global EGRA score | 2.41 | 0.07 | 2.04 | 0.05 | 2.42 | 0.01 | 2.52 | 0.03 |
| Whether child can read | 0.03 | 0.38 | 0.01 | 0.80 | 0.03 | 0.39 | 0.05 | 0.09 |
| Whether child can write | 0.01 | 0.87 | 0.05 | 0.19 | 0.07 | 0.03 | 0.04 | 0.21 |
| Highest schooling grade attained | 0.13 | 0.03 | 0.23 | 0.00 | 0.26 | 0.00 | 0.22 | 0.00 |
|  |  |  |  |  |  |  |  |  |
| *2013 survey (11–12 year olds):* |  |  |  |  |  |  |  |  |
| Height-for-age z-score (HAZ) | 0.05 | 0.57 | 0.05 | 0.48 | 0.09 | 0.24 | 0.18 | 0.06 |
| BMI-for-age z-score (BMIZ) | 0.05 | 0.55 | 0.19 | 0.05 | 0.11 | 0.21 | 0.31 | 0.00 |
| English percentage score (0–100) | **--** | -- | 1.70 | 0.24 | 3.05 | 0.03 | -- | -- |
| PPVT percentage score (0–100) | 2.06 | 0.15 | 4.06 | 0.00 | 3.62 | 0.00 | 1.79 | 0.05 |
| Language percentage score (0–100) | 2.25 | 0.13 | 0.62 | 0.64 | 1.50 | 0.23 | 2.71 | 0.05 |
| Mathematics percentage score (0–100) | 2.34 | 0.22 | 2.39 | 0.15 | 2.15 | 0.16 | 1.29 | 0.35 |
| Highest schooling grade attained | 0.24 | 0.10 | 0.25 | 0.01 | 0.22 | 0.01 | 0.25 | 0.00 |

Note: Data are from 2009 and 2013 rounds of the Young Lives survey—about 2,000 children each in the younger cohort in Ethiopia, India, and Vietnam. ATT denotes the propensity score matching estimator (kernel matching) of the association of measles vaccination as compared with BCG or polio immunization. Empty cells indicate outcome variables not measured in that survey round. Depending upon the country and survey round, some values of the outcome indicators were missing.

**References**

[1] Dehejia RH, Wahba S. Propensity Score-Matching Methods For Nonexperimental Causal Studies. The Review of Economics and Statistics 2002;84:151–61.

[2] Dehejia RH, Wahba S. Causal Effects in Nonexperimental Studies: Reevaluating the Evaluation of Training Programs. Journal of the American Statistical Association 1999;94:1053–62.

[3] Heckman JJ, Ichimura H, Todd PE. Matching as an Econometric Evaluation Estimator: Evidence from Evaluating a Job Training Programme. Review of Economic Studies 1997;64:605–54.

[4] Rosenbaum PR, Rubin DB. The central role of the propensity score in observational studies for causal effects. Biometrika 1983;70:41–55.

[5] Briones K. “How Many Rooms Are There in Your House?” Constructing the Young Lives Wealth Index. Young Lives, Oxford Department of International Development (ODID), University of Oxford; 2017.

[6] Rosenbaum PR, Rubin DB. Constructing a Control Group Using Multivariate Matched Sampling Methods That Incorporate the Propensity Score. The American Statistician 1985;39:33–8. doi:10.1080/00031305.1985.10479383.

[7] Sianesi B. An Evaluation of the Swedish System of Active Labor Market Programs in the 1990s. The Review of Economics and Statistics 2004;86:133–55.

[8] World Health Organization. Measles vaccines: WHO position paper – April 2017. Weekly Epidemiological Record 2017:205–28.

[9] Awofeso N, Rammohan A, Iqbal K. Age-appropriate vaccination against measles and DPT-3 in India - closing the gaps. BMC Public Health 2013;13:358–358. doi:10.1186/1471-2458-13-358.

[10] Abadie A, Drukker D, Herr JL, Imbens GW. Implementing Matching Estimators for Average Treatment Effects in Stata. The Stata Journal 2004;4:290–311. doi:10.1177/1536867X0400400307.

[11] WHO. World Health Organization Immunization Schedule. WHO Vaccine-Preventable Diseases: Monitoring System 2018 Global Summary 2018. http://apps.who.int/immunization_monitoring/globalsummary/schedules (accessed September 24, 2018).
